# Clinical Validation of Innovative Optical-Sensor-Based, Low-Cost, Rapid Diagnostic Test to Reduce Antimicrobial Resistance

**DOI:** 10.3390/jcm8122098

**Published:** 2019-12-01

**Authors:** Suman Kapur, Manish Gehani, Nagamani Kammili, Pankaj Bhardwaj, Vijayalakshmi Nag, Sudha M. Devara, Shashwat Sharad

**Affiliations:** 1 Department of Biological Sciences, Birla Institute of Technology and Science, Pilani, Hyderabad Campus, Hyderabad, Telangana 500078, India; dr.manishgehani@gmail.com; 2 Department of Microbiology, Gandhi Medical College and Hospital, Hyderabad, Telangana 500003, India; nagamaniy2k03@rediffmail.com (N.K.); sudha_devara@yahoo.com (S.M.D.); 3 All India Institute of Medical Sciences, Jodhpur, Rajasthan 342005, India; pankajbhardwajdr@gmail.com (P.B.); vijayalakshmi005@gmail.com (V.N.); 4 Center for Prostate Disease Research, Department of Surgery, Uniformed Services University of the Health Sciences and the Walter Reed National Military Medical Center, Bethesda, MD 20817, USA

**Keywords:** urinary tract infection, rapid culture, antibiotic susceptibility testing (AST), evidence-based prescription, antibiotics, antimicrobial resistance (AMR), rapid diagnostics

## Abstract

The antibiotic susceptibility test determines the most effective antibiotic treatment for bacterial infection. Antimicrobial stewardship is advocated for the rational use of antibiotics to preserve their efficacy in the long term and provide empirical therapy for disease management. Therefore, rapid diagnostic tests can play a pivotal role in efficient and timely treatment. Here, we developed a novel, rapid, affordable, and portable platform for detecting uropathogens and reporting antibiogram to clinicians in just 4 h. This technology replicates the basic tenets of clinical microbiology including bacterial growth in indigenously formulated medium, and measurement of inhibition of bacterial growth in presence of antibiotic/s. Detection is based on chromogenic endpoints using optical sensors and is analyzed by a lab-developed algorithm, which reports sensitivity to the antibiotic’s panel tested. To assess its diagnostic accuracy, a prospective clinical validation study was conducted in two tertiary-care Indian hospitals. Urine samples from 1986 participants were processed by both novel/index test and conventional Kirby Bauer Disc Diffusion method. The sensitivity and specificity of this assay was 92.5% and 82%, respectively (*p* < 0.0005). This novel technology will promote evidence-based prescription of antibiotics and reduce the burden of increasing resistance by providing rapid and precise diagnosis in shortest possible time.

## 1. Introduction

Healthcare challenges faced by developing countries are vastly different from those in developed nations. With very limited budget for healthcare, developing countries have not been able to put up any significant infrastructure to address their huge disease burden. In vitro diagnostic (IVD) tests provide the basis for most medical decision-making and play crucial role in limiting healthcare costs, since appropriate diagnostic tests performed in a timely manner i) improve patient care, ii) contribute to protecting consumers’ health, iii) help to limit healthcare spending, iv) reduce the risk of trial-and-error treatment and over-prescription, v) shorten the time before treatment begins, and vi) decrease the length of hospital stays. Appropriate diagnosis can improve the effectiveness of treatments and avoid long-term complications for the infected patient. India harbors the world’s largest burden of drug-resistant pathogens. Easy access, availability, and higher consumption of medicines have led to a disproportionately higher incidence of inappropriate use of antibiotics and greater levels of antimicrobial resistance (AMR) compared to developed countries [1]. It has been shown that the health sector in India suffers from gross inadequacy of funds, which will further result in conditions favorable for the development of drug resistance [2]. The high resistance of pathogens in the country, even to newer antibiotics, has led to the emergence of superbugs like New Delhi Metallo-beta-lactamase (NDM-1) [3]. By 2050, 2 million Indians are projected to die as a result of AMR [3]. Indians are the largest consumers of antibiotics worldwide, despite a decline in communicable diseases [3], due to a liberal policy for over-the-counter sale of antibiotics and irrational prescription of antibiotics. A study by Ganguly et al. highlighted the importance of rationalizing antibiotic use to limit AMR in India [4]. Irrational prescription happens due to a lack of fast point-of-use tests for evidence-based prescription, lack of infrastructure for bacterial culture and antibiotic susceptibility test (AST), and lack of awareness worldwide. Selective pressure from inappropriate use of antibiotics can lead to resistance via the emergence of mutant strains [5]. Unavailability of rapid point-of-use diagnostics to distinguish bacterial infections and suggest appropriate therapy is a major reason for irrational prescriptions of antibiotic/s. 

Urinary tract infections (UTIs) lead to 23% of all antibiotic prescriptions in primary healthcare. Even in India, UTIs account for about 8.1 million prescriptions each year. Diagnosis of UTI is a multistep process including determination of pathogen load, identification, and AST requiring culture of sample, which takes around 48–72 h. Even if high-throughput automated systems like Vitek 2, Microscan Walkaway, or Phoenix are used, the results are not available faster than 28 h [6,7]. Conventional urine culture and AST method is not accessible to most clinicians practicing in low-resource settings. Even with access to lab testing facilities, but in the absence of any rapid test, clinicians are forced to prescribe antibiotics empirically. The empirical antibiotics used in the first 48–72 h prove to be ineffective against infection in approximately 33% cases [8,9,10]. Unresolved, relapsed UTIs tend to be resistant to previously used antibiotics [11]. Nearly 23% to 33% of the prescriptions for UTIs have been found to have no clinical justification [8,9,10]. Moreover, UTIs are also caused by non-bacterial organisms such as Candida (3% cases) [12], Trichomonas (17% cases) [13], Chlamydia (~16% cases) [14], and rarely Mycobacterium, Schistosoma haematobium, Adenovirus, BK polyomavirus, and mycoplasma [15], and cannot be treated by antibiotics that are empirically prescribed. Such unnecessary drug use is often harmful, and results in multidrug-resistant infections and reduced options for antimicrobial therapy [16]. An urgent need is perceived for developing suitable field operable test for prescribing targeted antibiotics [17]. Addressing the menace of antimicrobial resistance needs a scalable rapid diagnostic test, which gives detection, identification, quantification, and phenotypic antimicrobial susceptibility of bacteria within a minimum turn-around time and has an integrated technology platform for clinical adoption. This test should show high sensitivity and specificity, should be low cost for adoption in low-resource settings, and should be easy to use with minimal training [18,19]. 

Assays used for upstream screening to improve diagnostic yield of positive samples, like gram staining, dipstick with leukocyte esterase and nitrite, pus cell count [20], urine analysis and microscopy [21], chlorhexidine [22], interleukin-8 [23], Griess test [24], microstix [25], serum procalcitonin level [26], and urine catalase-based uriscreen test [27] have shown poor sensitivity and specificity. Novel antibody-based lateral flow immunoassay (RapidBac) [28], chromogenic limulus amoebocyte lysate assay [29], and flow cytometry-based systems (Accuri-6, UF 100, UF-1000i) [18,30,31] have shown high sensitivity and specificity but they do not provide identification of bacteria and its antimicrobial susceptibility. Forward light scattering systems like Uro-Quick (Alifax) and BacterioScan model 216 (BacterioScan Inc., St. Louis, MO, USA) provide detection of bacteria with antimicrobial susceptibility but do not identify the causative bacteria [32,33]. 

Molecular and proteomic technologies require overnight incubation on culture plates, and do not provide antimicrobial susceptibility [18]. Matrix Assisted Laser Desorption Ionization-Time Of Flight (MALDI-TOF) is expensive to install [34], Fluorescence In Situ Hybridization (FISH) requires multiple probes for all uropathogens [18], while multiplex Polymerase Chain Reaction (PCR) platforms like GeneXpert Omni and Cepheid [18] do not provide quantification of significant bacteriuria and need multiple probes for all uropathogens. Application of these test to direct urine testing needs extensive sample preparation. 

Genetic signature identification Confirming Active Pathogens Through Unamplified RNA Expression (CAPTURE) assay [35] identifies bacteria but does not give antimicrobial susceptibility. Time-lapse microscopy-based systems like oCelloScope (Phillips BioCell) [36] and Accelerate ID/AST (Accelerate Diagnostics) [37] provide both identification and antimicrobial susceptibility, but phenotypic measures for identification in direct urine are not precise and they are not easy to use in a clinical lab setting. Integrated microfluidic-Biosensor assays based on ion mobility spectrometry or colorimetric sensor arrays are cost effective and sensitive but their results get confounded by urine variability and in presence of low bacterial count [18]. Most of the newer technologies mentioned above are neither easy to use nor affordable in a resource-poor setting like public hospitals of India. In this study, we evaluated a rapid, portable, easy-to-use, less resource-intensive, and affordable technology, which provides bacterial identification and AST results within 4 h. This new technology integrates the basic tenets of clinical microbiology including bacterial growth in a medium optimized for uropathogens and measurement of inhibition of bacterial growth in presence of specific antibiotic, with detection of bacteria based on chromogenic endpoint by enzymatic hydrolysis of specific media cocktails by UTI causing bacteria. The optical sensor-based measurement of endpoint output is analyzed using indigenous software, based on a lab-developed statistical algorithm, which reports both the sensitivity of the pathogen to a customizable panel of antibiotics and bacterial load in the sample. This integrated technology platform can be used for diagnosing UTIs caused by bacteria and for suggesting effective antibiotics in all types of clinical settings as a preliminary triage test [38] to promote evidence-based prescription and minimize irrational use of antibiotics. The low cost of the test obliviates the need for upstream screening with poor sensitivity screening tests and promotes scalability for use in mass population. The objective of the present study was to evaluate the diagnostic accuracy of the novel test in UTI cases as compared to the gold standard urine culture method.

## 2. Materials and Methods

### 2.1. Study Design, Setting, and Population

The study was conducted over a 2-year period from January 2017 to December 2018, simultaneously in Gandhi Medical College and Hospital, Secunderabad located in Southern India, and All India Institute of Medical Sciences (AIIMS), Jodhpur located in Northern India. To ensure sufficient case load for achieving required sample size and to ensure that good lab practices are followed, Laboratory of Gandhi Hospital, which is the referral laboratory of State of Telangana, and AIIMS, which is a premiere tertiary care hospital, were chosen for this study. 

### 2.2. Ethical Approval 

The study was reviewed and approved by Institutional Ethics Review Committee of both institutions. Objectives of the study were explained to all participants in their native language and they were enrolled after obtaining a written informed consent. The study was conducted according to the principles expressed in the Declaration of Helsinki. 

### 2.3. Study Oversight 

This prospective clinical validation study was designed to evaluate diagnostic accuracy of the novel/index test with the reference gold standard urine culture and AST method. Eligible participants were referred by clinicians for urine culture and sensitivity test, based on a provisional diagnosis of UTI. Patients who received antibiotics in the preceding two weeks or had indwelling or suprapubic catheter were excluded. Consenting participants were evaluated in microbiology laboratory by history taking and review of medical records.

### 2.4. Test Methods for Bacterial Culture and Identification

Clean-catch mid-stream urine samples were collected from each enrolled participant in a sterile container and divided into two parts under sterile conditions. One part was used for routine urine culture and AST and the second for conducting the index test in the hospital premises itself. All samples were processed within 2 h of collection to avoid contamination/bacterial growth.

The index test was the novel test designed for direct quantitative detection and antibiotic sensitivity of bacteria found in human urine [39,40]. The test identifies common UTI-causing bacteria, namely *Escherichia coli*, *Klebsiella*, *Pseudomonas*, *Enterococcus*, *Proteus*, and *Staphylococcus* sp. This rapid method replicates the basic tenets of clinical microbiology, namely (1) growth of bacteria in a specialized medium, and (2) measuring the inhibition of growth of bacteria in the presence of an antibiotic. Detection is based on chromogenic endpoints. The output was analyzed using lab-developed algorithm-based software, which reports the sensitivity of the pathogen to the panel of antibiotics tested. The urine sample was collected in a sterile container. To harvest the bacteria, 10 mL urine was filtered through a sterile syringe with the help of a micro-filter attached to it and filtrate was discarded. After that, BITGEN, specially designed media for accelerated growth of uropathogens, was pushed through the filter in the vial to recover bacteria from the filter, shaken well, and then closed with the dropper cap. The bacteria were harvested in 3 mL of proprietary BITGEN medium. This was then set side at room temperature for about 5 min. Subsequently, four drops (~110–120 μL) of proprietary BITGEN medium containing harvested bacterial suspension was added into all the three strips—one pre-functionalized strip for identification of bacteria and two different 8-well strips, pre-loaded with antibiotics. All the strips were resealed and incubated at 37 °C for 4 h. A 4-h incubation period was found to be sufficient for all commonly found uropathogens accounting for 98% cases of UTIs. The media was optimized for nutrients and supports growth up to 8 h with a start bacterial number of 10^5^ cells/mL [39]. BITGEN is a proprietary media that has chromogens sensitive to bacterial growth even at low numbers of bacteria and for rapid culture. The enzymatic hydrolysis of specific media cocktails used in this proprietary media metabolizes the chromogens. For identification of bacteria, the 8 wells in the identification strip had a cocktail of specific substrates, which were metabolized by specific bacterial types. Growth of bacteria in the well led to end product formation during the 4-h incubation. The use of optical sensor enables measuring of all color combinations and the lab-developed analytical software interprets the identification of the bacteria based on specific chromogenic endpoints produced as a consequence of specific metabolic activity of each bacterial type. For both identification and AST, the sample was loaded at the same time and incubated for the same length of time. 

To identify susceptibility of pathogen, the above-mentioned two pre-functionalized antibiotic strips were used. Each of the antibiotic strips had 8 compartments and, except the first compartment (or reference well) of each of the two antibiotic strips, all the remaining 14 compartments were subjected to preloading by the chosen antibiotics. The preloaded antibiotics used were Amoxicillin, Gentamicin, Amikacin, Cefepime, Ofloxacin, Ciprofloxacin, Ceftriaxone, Piperacillin-Tazobactum, Cefotaxime, Cefuroxime, Tobramycin, Levofloxacin, Cefazolin, and Imipenem. The concentration and composition of the antibiotics were chosen as per Clinical and Laboratory Standards Institute (CLSI) guidelines [41].

In the case that the urine sample had pathogens, it was reflected in the first well of the antibiotic strips, referred to as the reference well of both the antibiotic strips as there is no inhibition of bacterial growth in this well. As per phenotypic AST of bacteria present, the remaining 14 compartments showed varied levels of bacterial growth depending on the bacterial susceptibility to the chosen antibiotics. The bacterial growth within the preloaded antibiotic compartment was represented by a change in color of the BITGEN, measured by chromogenic and nephelometric endpoints using an array of 64 photodiodes in an electronic optical sensor. The intensity of the color is a measure of the number of growing cells in the presence and absence of a particular antibiotic. The sensor output was analyzed using a proprietary lab-developed statistical algorithm, pre-installed on the reader, which provides ready-to-use results for sensitivity of the pathogen to the antibiotics tested, both as a display on liquid crystal display (LCD) screen and a printout for permanent records. The reader was also enabled to transfer results to other storage devices using a wireless module and/or a universal serial bus (USB) interface. In case of insufficient growth, the analytical software prompts for incubating for one additional hour and then if no growth is detected, the software reports the sample to be negative for presence of bacteria.

Further, for reference, standard universally accepted, conventional gold standard urine culture and Kirby Bauer method for AST was chosen. First, 10 μL of each urine sample was streaked on a chromogenic culture medium, chromID^®^ CPS Elite Translucent using a calibrated ni-chrome wire loop of 4 mm by semi-quantitative method using surface streaking. The inoculated plates were incubated for 18–24 h at 37 °C. After incubation, in case growth of colonies was up to the tertiary streaking, it was considered as significant bacteriuria with 10^5^ Colony Forming Units (CFU)/mL. Positive cultures were further processed for determining the AST by Kirby Bauer Disc Diffusion Method as per Clinical and Laboratory Standards Institute guidelines [41]. A suspension of each isolate was prepared to a McFarland standard and spread over Muller Hilton Agar using lawn culture method. Himedia discs with defined concentrations of antibiotics were placed over the culture. After incubation for 18 to 24 h at 37 °C, zones of growth inhibition around each antibiotic disc were measured to the nearest millimeter and a reference table was used to determine susceptibility. The American Type Culture Collection (ATCC) bacterial strains, namely *Enterococcus faecalis*, *Escherichia coli*, *Klebsiella pneumoniae*, *Pseudomonas aeruginosa*, and *Staphylococcus aureus*, were used for quality control in the entire process.

The cut-off for labelling both index as well as reference test as positive was pre-specified as 10^5^ CFU/mL based on Infectious Diseases Society of America guidelines [42]. Neither the team performing the index test nor the one conducting urine culture and sensitivity was provided any clinical information about the participant/s. Both teams were also not informed about the results of the other test and, hence, the index test was conducted in a completely blinded manner.

### 2.5. Data Analysis

Collected data and results from both tests for each participant were compiled and analyzed by Statistical Package for the Social Sciences (SPSS) software (version 24). A contingency table was used for determining diagnostic accuracy and kappa statistics was used for agreement analysis. Further, 95% confidence interval (CI) was used to describe diagnostic accuracy, with *p* values of <0.05 considered as significant. Sample size was calculated to be 600 for estimating the sensitivity of the index test, based on a precision of 4% and confidence level of 95%, when the sensitivity of the new test was expected to be at least 50%. “Best-case scenario method” was used for indeterminate results and mixed growth. Samples with rare species, budding yeast cells, and contaminated samples were removed from final analysis for a “complete case analysis”. No analysis of variability in diagnostic accuracy was performed with respect to age group or department, as it was not pre-specified in the study. The raw data generated from the study which was used to analyze these results has been made publicly available as a safe harbor file in online repository “Harvard Dataverse” [43].

### 2.6. Reagents

Analytical-grade chemicals required for preparation of BITGEN, identification strips, and antibiotic strips were procured from Sigma Chemicals, St Louis, MO, USA. Chromogenic culture media, Muller Hilton media, and antibiotic discs were procured from Himedia, India; chromID^®^ CPS Elite Translucent from BioMérieux, France; 8-well strips and syringe filters from NUNC, Denmark; sterile syringes from Dispovan, India. The scanner/reader machine for novel test was obtained from Micro Lab Instruments, Ahmedabad, India. The bacterial strains *Enterococcus faecalis* (ATCC29212), *Escherichia coli* (ATCC25922), *Klebsiella pneumoniae* (ATCC13883), *Pseudomonas aeruginosa* (ATCC27853), and *Staphylococcus aureus* (ATCC25923) were purchased from Himedia, India.

## 3. Results

### 3.1. Study Characteristics

Overall, 2001 eligible participants (1030 in AIIMS and 971 in Gandhi Hospital) were identified and 1986 participants (1022 in AIIMS and 964 in Gandhi Hospital) were enrolled in the study. Data of 1835 participants (982 in AIIMS and 853 in Gandhi Hospital) were included in the final analysis. A total of 55 samples (20 from AIIMS and 35 from Gandhi Hospital) with low sample volume could not be processed by the index test.

**Figure 1:**
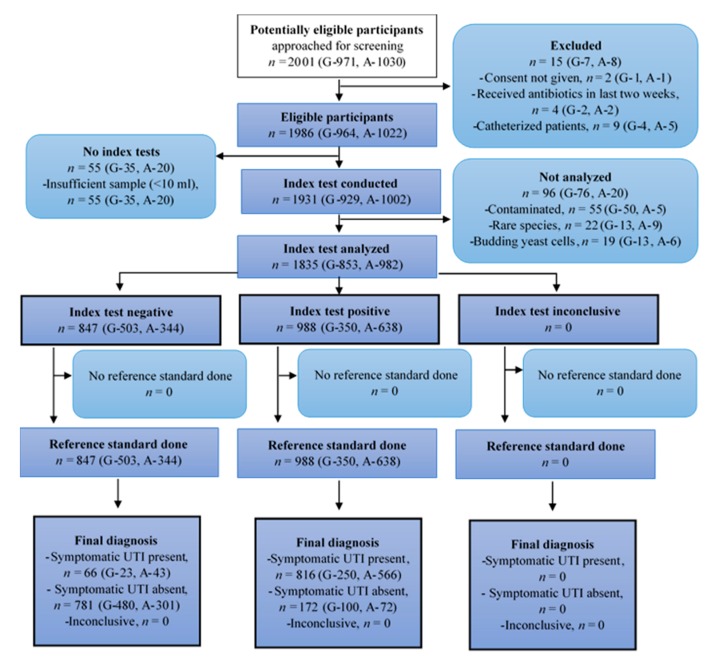
Flow of participants through the study—Standards for Reporting Diagnostic Accuracy (STARD) diagram (G = Gandhi Hospital, A = AIIMS).

There were no indeterminate results reported by the index test in both the hospitals. One hundred and eleven participants (97 in AIIMS and 14 in Gandhi Hospital) with indeterminate reference standard urine culture results were reported as having no bacterial growth and were reclassified as true negatives using best-case scenario. Samples with mixed growth in both index and reference standard tests were considered positive for UTI. Fifty-five samples (5 from AIIMS and 50 from Gandhi Hospital) were reported as contaminated and not considered for final analysis. Since the index test is designed for identifying the most common bacteria only, 19 samples (6 from AIIMS and 13 from Gandhi Hospital) with budding yeast cells and 22 samples (9 from AIIMS and 13 from Gandhi Hospital) with rare bacteria (*Citrobacter*, *Acinetobacter*, *Morganella*, and *Providencia*) were also excluded from the final analysis. Thus, a total of 96 cases were excluded from final analysis after performing both tests (Figure 1). Table 1 summarizes the mean age, gender distribution, and referring departments. The majority of participants had cystitis, and more male patients were referred at AIIMS than Gandhi Hospital. Ninety-seven cases (10%) cases in AIIMS cohort and nine cases (1%) in Gandhi Hospital cohort had progressed to frank pyelonephritis.

### 3.2. Test Performance 

There was no time gap between processing of samples by both tests. No adverse event occurred while performing index test or reference standard test since only urine sample collection was involved. In AIIMS, 609 cases, while in Gandhi Hospital, 273 cases, were diagnosed with symptomatic UTI based on positive culture results by conventional method. Furthermore, 953 participants (373 in AIIMS and 580 in Gandhi Hospital) with symptoms of UTI, showed low colony count on culture plates. Out of these, the index test reported 172 cases (72 in AIIMS and 100 in Gandhi Hospital) as positive, which were otherwise reported as negative by conventional method, 48 h post incubation. 

#### 3.2.1. Diagnostic Accuracy

AIIMS cohort showed a higher sensitivity (92.9%) while Gandhi Hospital cohort showed a marginally higher specificity (82.8%). The sensitivity and specificity in both the validation sites were within 95% confidence interval of the other hospital (Table 2). The sensitivity and specificity obtained by use of the novel test was well within the stipulated limits laid down in the recommendations issued by the European Urinalysis Guidelines for rapid tests.

#### 3.2.2. Agreement Analysis 

Good agreement was observed at both validation sites, as seen by a Kappa = 0.741. The observed agreement is statistically significant as reflected by a *p* value of <0.0005. 

### 3.3. Identification of Bacteria 

The index test correctly reported the causative bacteria as reported positive by urine culture in 82% cases in AIIMS cohort and 80% cases in Gandhi Hospital cohort (Table 3). In Gandhi Hospital, four cases of *Streptococcus* were not reported by the index test as it is not designed to identify the same. Out of 17 mixed growth in Gandhi cohort, the index test identified seven as individual bacteria, while among 49 in AIIMS cohort, it identified 41 as individual bacteria. 

### 3.4. Antibiotic Susceptibility 

The index test used the same set of 14 antibiotics for every sample, while AIIMS and Gandhi Hospital laboratories used specific antibiotics based on identified bacteria. Hence, only a subset of antibiotics overlapped for both tests. Further, as the conventional method relied on the choice of antibiotics by the microbiologist in-charge, sets of antibiotics tested in both tests were also not used for all the samples tested. Antibiotics tested for at least 30 samples in both the tests were included in analysis presented in Table 4. The rapid index test correctly reported sensitivity and resistance to antibiotics in 91% and 96% cases, respectively, in AIIMS cohort, and these numbers were 87% in the case of sensitivity to tested antibiotics and 92% in the case of resistance to antibiotics reported for the Gandhi Hospital cohort. 

## 4. Discussion

Treating patients, including UTIs caused by bacteria, is a challenging task, and development of rapid AST is very important to provide better healthcare services. Use of microbiological culture method and Kirby–Bauer disc diffusion tests are well established for diagnosis of UTIs in healthcare facilities worldwide. However, this entire method needs trained microbiologists and its major limitations are long turn-around time, resource intensiveness in the form of lab infrastructure, and requirement of cold chain for supply and storage of reagents [44]. In resource-constrained settings with poor or limited access to laboratory-based testing, performing urine culture and AST is not feasible. Therefore, initial antibiotic therapy in infectious diseases such as UTIs which accounts for ~40% cases of all infections as per World Health Organization (WHO), is mostly empirical. Hence, an alternative method, like the index test described herein, for reporting antibiotic sensitivity in a short period of 4 h, with no ancillary resource requirement, will not only be beneficial for patient care, but also curtail unnecessary antibiotic prescriptions. Additionally, availability of results in 4 h saves the repeat visit of patients to collect lab reports made available only after three days under best conditions and often even longer in remote and hard-to-reach geographical locations.

The high-cost, resource-intensive, non-portable, most commonly used automated systems Vitek 2 and MicroScan Walkaway provide AST and identification results in more than 28 h [6,7]. Reports evaluating the susceptibility of only Gram-negative bacilli to 11 antibacterial using these two systems showed the results in 92.7% of isolates and overall concurrence with the standard test being 94% with a 3.4% major error rate [45]. With reference to preventing emergence of resistance to antibiotics, they still pose a major limitation in terms of time taken to complete the identification and antibiogram profile of UTI causing pathogen. Most of the newer technologies tried for UTI [18] are facing limitations like the need for an overnight incubation, extensive initial sample preparation, need for an upstream screening test, and lack of integrated technology platform for clinical adoption. These technologies are expensive and not easy to use. Most of them do not give antimicrobial susceptibility. Previously tried strip-based tests also showed less sensitivity [46]. Even automated urine analyzers have resulted in low sensitivity [47]. In comparison, this index test is portable, can be used in all healthcare settings, costs less than 0.4 million INR (~5000 USD), needs no ancillary equipment or dedicated space, and provides ready-to-use antibiogram results and microbial identification within 4 h. The sensitivity and limitation of other tests are summarized in Table 5. The higher sensitivity, >90%, and specificity, >80%, of the index test, with kappa values indicating very good agreement with gold reference standard test, show that it has good diagnostic accuracy as a rapid test [48] for its role as a preliminary triage test and its intended use of diagnosing bacteriuria and preventing irrational prescription of antibiotics. Although the gold standard for diagnosing UTI remains as urine culture, the high cost, laboratory requirements, and long turnaround times (24–72 h) are its disadvantages. Further, in this type of testing, recognition and classification of bacteria is associated with the experience of laboratory technicians. The novel/index test developed is a simpler system and shows better agreement (Kappa = 0.741, significant substantial agreement) with the gold standard and is therefore best suited for routine use in clinical laboratories.

This novel test correctly identified sensitivity to multiple antibiotics in more than 75% instances (and in several cases with 100% accuracy), which then becomes the basis for evidence-based, rational use of antibiotics for specific therapy. The availability of results within 4 h will discourage unnecessary prescription of antibiotics in case of absence of bacterial disease and help the physician to prescribe antibiotics that are identified to be effective against the causative pathogen.

The incidence of UTI from this hospital-based study may not be generalized over the entire population as the present study was conducted in tertiary hospitals to enroll enough participants in the shortest possible time and simultaneous comparison with reference gold standard test without any loss of time in processing or transport of collected samples. Due to the conventional practice in microbiology labs on the choice of antibiotics, which may also be governed by the availability of antibiotics discs, antibiotics could be compared for sensitivity in a subgroup of samples tested. In spite of this, the novel test reported resistance to antibiotics with an accuracy of 80% to 100%, except for two antibiotics, cefazolin (71%) and levofloxacin (65%). This was primarily seen in samples with more than one bacterial entity with a high probability of quorum sensing, impacting the sensitivity to the given antibiotic/s. 

Rapid assay described herein determines the efficacy of an antibiotic not only in the shortest possible time, but also with literally no dependence on trained manpower and lab infrastructure. Although this novel test is suitable for use in all healthcare settings, it can prove to be of immense and unsurpassable value for healthcare facilities in low-resource settings due to features like portability, point-of-use testing, and no additional requirements. A consensus using Delphi technique, obtained from experts regarding criteria required for an acceptable point-of-care test for UTI detection, was reported by Weir et al. [49]. This novel test fulfils 25 out of 26 accepted criteria, except for just one, i.e., use of small sample volume. The novel test also fulfils six out of seven of the WHO’s ASSURED criteria for ideal characteristics for a point-of-care test in resource-limited settings, the sole unfulfilled one being equipment free, and also matches all the revised criteria suggested by Paul et al. [50]. Effective diagnosis is a prerequisite for successful therapy, and early and accurate diagnosis results in timely and appropriate treatment.

## 5. Conclusions

In conclusion, it can be said that this novel test, with high sensitivity and specificity for detecting bacterial UTI and reporting antibiogram, can be used as a triage test for diagnosing UTIs and suggesting appropriate treatment for an evidence-based prescription for antibiotics in any kind of healthcare settings. In the wake of growing AMR, the prevalent “lack of priority for diagnostics over treatment” needs to be addressed urgently and this novel test enables physicians and labs to achieve this by adopting this affordable and portable IVD test before prescribing antibiotics for treatment of infectious diseases.

## Figures and Tables

**Table 1:** Characteristics of participants.

Characteristics	AIIMS ^1^ (*n* = 982)	Gandhi (*n* = 853)
**Demographic characteristics**		
**Age (in years)**		
Mean age	43.4	35.7
Minimum age	<1	1
Maximum age	95	90
**Gender**		
Male	631 (64.3%)	399 (46.8%)
Female	351 (35.7%)	454 (53.2%)
**Referring Department**		
Medical Specialties	193	481
Surgical Specialties	632	94
Pediatrics	67	138
Obstetrics and Gynecology	87	140
Radio-diagnosis	3	0
**Clinical Syndrome**		
Pyelonephritis	10%	1%
Cystitis	90%	99%

^1^ AIIMS = All India Institute of Medical Sciences.

**Table 2:** Comparison of test results obtained by novel test and urine culture method.

Contingency Tables			
**AIIMS (*n* = 982)**	**Urine Culture Positive**	**Urine Culture Negative**	**Total**
Index test Positive	566 (92.9%)	72 (19.3%)	638
Index test Negative	43 (7.1%)	301 (80.7%)	344
Total	609	373	982
**Gandhi (*n* = 853)**	**Urine Culture Positive**	**Urine Culture Negative**	**Total**
Index test Positive	250 (91.6%)	100 (17.2%)	350
Index test Negative	23 (8.4%)	480 (82.8%)	503
Total	273	580	853
**Combined (*n* = 1835)**	**Urine Culture Positive**	**Urine Culture Negative**	**Total**
Index test Positive	816 (92.5%)	172 (18.0%)	988
Index test Negative	66 (7.5%)	781 (82.0%)	847
Total	882	953	1835
**Diagnostic Accuracy**			
**Parameters**	**AIIMS (*n* = 982)**	**Gandhi (*n* = 853)**	**Combined (*n* = 1835)**
Sensitivity	92.9% (95% CI: 90.6–94.8%)	91.6% (95% CI: 87.6–94.6%)	92.5% (95% CI: 90.6–94.2%)
Specificity	80.7% (95% CI: 76.3–84.6%)	82.8% (95% CI: 79.4–85.8%)	82.0% (95% CI: 79.4–84.3%)
**Agreement Analysis**			
**Parameters**	**AIIMS (*n* = 982)**	**Gandhi (*n* = 853)**	**Combined (*n* = 1835)**
Kappa value ^1^	0.748	0.692	0.741
Standard error ^1^	0.022	0.025	0.016
*p* value	<0.0005	<0.0005	<0.0005

^1^ Kappa value and its standard error measures agreement between results of two dichotomous variables (here two diagnostic tests providing positive or negative results).

**Table 3:** Identification of bacteria in the two cohorts.

Identification of Bacteria (Single Species Identification)							
**AIIMS**	***E. coli***	***Enterococcus***	***Klebsiella***	***Proteus***	***Staphylococcus***	***Pseudomonas***	**Total**
“*n*” (based on urine culture)	324	104	79	4	1	48	560
% correct identification by index test	93%	74%	68%	75%	100%	48%	82%
**Gandhi**	***E. coli***	***Enterococcus***	***Klebsiella***	***Proteus***	***Staphylococcus***	***Pseudomonas***	**Total**
“*n*” (based on urine culture)	92	22	100	8	22	8	252
% correct identification by index test	85%	82%	83%	63%	50%	88%	80%

**Table 4:** Comparison of antibiotic susceptibility report in the two cohorts using both tests.

**AIIMS**				
	**AIIMS Result**	**Total Tests Done Together**	**Agreement in Results**	**Disagreement in Results**
**Gentamycin**	R	149	141 (95%) ^a^	8 (5%) ^b^
	S	259	237 (92%) ^a^	22 (8%) ^c^
	I	7	0	7 ^d^
**Amikacin**	R	65	65 (100%) ^a^	0 ^b^
	S	29	26 (90%) ^a^	3 (10%) ^c^
	I	3	0	3 ^d^
**Ciprofloxacin**	R	49	43 (88%) ^a^	6 (12%) ^b^
	S	1	1 (100%) ^a^	0 ^c^
	I	0	0	0 ^d^
**Ceftriaxone**	R	269	263 (98%) ^a^	6 (2%) ^b^
	S	99	85 (86%) ^a^	14 (14%) ^c^
	I	4	0	4 ^d^
**Piperacillin-Tazobactum**	R	121	118 (98%) ^a^	3 (2%) ^b^
	S	286	266 (93%) ^a^	20 (7%) ^c^
	I	22	0	22 ^d^
**Cefazolin**	R	34	24 (71%) ^a^	10 (29%) ^b^
	S	12	10 (83%) ^a^	2 (17%) ^c^
	I	0	0	0 ^d^
**Imipenem**	R	56	56 (100%) ^a^	0 ^b^
	S	21	19 (90%) ^a^	2 (10%) ^c^
	I	3	0	3 ^d^
**Overall**	R	743	710 (96%) ^a^	33 (4%) ^b^
	S	707	644 (91%) ^a^	63 (9%) ^c^
**Gandhi**				
	**Gandhi Result**	**Total Tests Done Together**	**Agreement in Results**	**Disagreement in Results**
**Gentamycin**	R	56	54 (96%) ^a^	2 (4%) ^b^
	S	120	103 (86%) ^a^	17 (14%) ^c^
	I	0	0	0 ^d^
**Amikacin**	R	12	12 (100%) ^a^	0 ^b^
	S	27	23 (85%) ^a^	4 (15%) ^c^
	I	0	0	0 ^d^
**Cefepime**	R	35	34 (97%) ^a^	1 (3%) ^b^
	S	10	8 (80%) ^a^	2 (20%) ^c^
	I	0	0	0 ^d^
**Piperacillin-Tazobactum**	R	13	12 (92%) ^a^	1 (8%) ^b^
	S	21	20 (95%) ^a^	1 (5%) ^c^
	I	0	0	0 ^d^
**Cefotaxime**	R	28	28 (100%) ^a^	0 ^b^
	S	14	14 (100%) ^a^	0 ^c^
	I	0	0	0 ^d^
**Levofloxacin**	R	23	15 (65%) ^a^	8 (35%) ^b^
	S	8	6 (75%) ^a^	2 (25%) ^c^
	I	0	0	0 ^d^
**Cefazolin**	R	56	50 (89%) ^a^	6 (11%) ^b^
	S	20	18 (90%) ^a^	2 (10%) ^c^
	I	0	0	0 ^d^
**Overall**	R	223	205 (92%) ^a^	18 (8%) ^b^
	S	220	192 (87%) ^a^	28 (13%) ^c^

R = Resistant; S = Sensitive; and I= Intermediate; a—complete agreement, b—very major error, c—major error, and d—minor error. (Same test results either susceptible or resistant by both tests, were classified as “complete agreement” and result reported as resistant by culture and susceptible by novel test was labelled as “very major error”; susceptible by culture but resistant by novel test was labelled as “major error”; intermediate by culture and susceptible or resistant by novel test was labelled as “minor error”). Please note that no intermediate results were reported by novel test and by Gandhi Hospital culture reports.

**Table 5:** Accuracy of different methods for estimating presence of bacteria.

Sl. No.	Method	Sensitivity	Specificity	Detection of Bacteriuria	Identification of Bacteria	Antibiotic Susceptibility	Limitations	Reference
1	MALDI-TOF	67% to 86%	Nearly 60%	Yes	Yes	No	Overnight incubation needed, expensive, extensive sample preparation	[18]
2	FISH	>96%	>96%	Yes	Yes	No	Requires multiple probes for all pathogens	[18]
3	PCR	82%	60%	Yes	Yes	No	Does not provide quantification, needs extensive initial processing and multiple probes	[18]
4	Integrated microfluidics-biosensor systems	91% to 95%	95% to 99%	Yes	Yes	Yes	Confounded results by urine variability and low bacterial count	[18]
5	Gram staining	85.1%	98.9%	Yes	No	No		[20]
6	Dipstick with nitrite & leucocyte esterase	53.1%	100%	Yes	No	No		[20]
7	Pus cell count	42.5%	95.5%	Yes	No	No		[20]
8	Urine analysis and microscopy	46.4%	89%	Yes	No	No		[21]
9	Chlorhexidine	100%	54%	Yes	No	No		[22]
10	Interleukin-8	70%	67%	Yes	No	No		[23]
11	Griess test	63.3%	99.5%	Yes	No	No		[24]
12	Serum procalcitonin level	30%	100%	Yes	No	No		[26]
13	Uriscreen Test	100%	68.6%	Yes	No	No		[27]
14	Antibody-based Lateral flow immunoassay	86%	94%	Yes	No	No		[28]
15	Chromogenic amoebocyte lysate assay	88.7%	98.7%	Yes	No	No		[29]
16	Flow cytometry-based systems	99%	58%	Yes	No	No		[30,31]
17	Genetic signature identification CAPTURE assay	100%	90%	Yes	Yes	No	Expensive, resource intensive, infrastructure, highly skilled manpower	[35]
18	Time-lapse microscopy	96% (agreement)	96% (agreement)	Yes	Yes	Yes	Imprecise phenotypic identification measures in direct urine, not easy-to-use	[36,37]
19	Kirby Bauer Method	51%	99%	No	No	Yes	Time-consuming, resource intense, and not user-friendly	[41,42]
20	Semiquantitative culture method	72.7%	95.7%	Yes	Yes	Yes	Time-consuming, resource intense, and not user-friendly	[44]
21	Quantitative culture method	59.3%	94.4%	Yes	Yes	Yes	Time-consuming, resource intense, and not user-friendly	[44]
22	Strip-based Urinalysis DongJiu	31.1%	91.8%	Yes	No	No		[46]
23	Automated Urinalysis system (URISED)	47%	91.1%	Yes	No	No		[47]
24	Detection of bacteriuria by a non-culture method	90% to 95%	90% to 95%	Not Applicable	Not Applicable	Not Applicable	European Guidelines for Urinalysis	[48]
25	Detection of bacteriuria by a rapid non-culture method	80% to 90%	80% to 90%	Not Applicable	Not Applicable	Not Applicable	European Guidelines for Urinalysis	[48]
26	Index Test	91.6 to 93.8%	80.7 to 96.7%	Yes	Yes	Yes	User-friendly, portable, affordable, rapid-fastest available, no special training required	Data from current study described above
